# Synthesis, Crystal Structures, Hirshfeld Surface Analysis, Computational Investigations, Thermal Properties, and Electrochemical Analysis of Two New Cu(II) and Co(II) Coordination Polymers with the Ligand 5-Methyl-1-(pyridine-4-yl-methyl)-1H-1,2,3-triazole-4-carboxylate

**DOI:** 10.3390/ijms26041671

**Published:** 2025-02-15

**Authors:** Markus Bergedahl, Pilar Narea, Jaime Llanos, Ruth Pulido, Nelson Naveas, Pilar Amo-Ochoa, Félix Zamora, Gerzón E. Delgado, Felipe M. Galleguillos Madrid, Yasna León, Iván Brito

**Affiliations:** 1 Departamento de Química, Facultad de Ciencias Básicas, Universidad de Antofagasta, Casilla 170, Antofagasta 1240000, Chile; markus.bergedahl.fredes@ua.cl (M.B.); pilar.narea@uantof.cl (P.N.); ruth.pulido@uantof.cl (R.P.); yasna.leon@uantof.cl (Y.L.); 2 Departamento de Química, Facultad de Ciencias, Universidad Católica del Norte, Avda. Angamos 0610, Antofagasta 1270709, Chile; jllanos@ucn.cl; 3 Instituto Universitario de Ciencia de Materiales “Nicolás Cabrera” (INC), Universidad Autónoma de Madrid, Campus de Cantoblanco, 28049 Madrid, Spain; nelson.naveas@estudiante.uam.es; 4 Departamento de Física Aplicada, Universidad Autónoma de Madrid, 28049 Madrid, Spain; 5 Departamento de Ingeniería Química y Procesos de Minerales, Universidad de Antofagasta, Avenida Angamos 601, Antofagasta 1270300, Chile; 6 Departamento de Química Inorgánica, Universidad Autónoma de Madrid, 28049 Madrid, Spain; pilar.amo@uam.es (P.A.-O.); felix.zamora@uam.es (F.Z.); 7 Institute for Advanced Research Chemistry (IAdChem), Universidad Autónoma de Madrid, 28049 Madrid, Spain; 8 Condensed Matter Physics Center (IFIMAC), Universidad Autónoma de Madrid, 28049 Madrid, Spain; 9 Laboratorio de Cristalografía, Departamento de Química, Facultad de Ciencias, Universidad de Los Andes, Mérida 5101, Venezuela; gerzon@ula.ve; 10 Centro de Desarrollo Energético de Antofagasta, Universidad de Antofagasta, Av. Universidad de Antofagasta 02800, Antofagasta 1240000, Chile; felipe.galleguillos@uantof.cl

**Keywords:** coordination polymers, 1,2,3-triazole, metal–organic framework, electrochemical properties

## Abstract

Two new Cu(II) (CP1) and Co(II) (CP2) coordination polymers (CPs) with the triazole ligand 5-methyl-1-(pyridin-4-yl-methyl)-1H-1,2,3-triazole-4-carboxylate (L1) have been synthesized and structurally characterized by SCXRD (Single Crystal X-Ray Difraccion), PXRD (Power X-Ray Difracction), FT-IR (Fourier Transform Infrared), TG (Theermo Gravimetric), and electrochemical techniques. Both CPs were obtained at the water/n-butanol interface by reacting nitrate salts of each metal with the NaL1 ligand. SCXRD analysis revealed that CP1 (Coordination Polymer 1) and CP2 (Coordination Polymer 2) crystallize in the monoclinic space groups C2/c (No. 15) and P2_1_/n (No. 14), respectively, forming 1D zigzag chain structures, which further lead to a 2D supramolecular network through O-H⋯O and C-H⋯O hydrogen bond interactions, respectively. In CP1, the supramolecular structure is assembled by hydrogen bonds involving water molecules. In contrast, CP2 forms its supramolecular network mainly through hydrogen bonds between adjacent triazole ligand molecules. Hirshfeld surface analysis revealed that the most significant contributions to the crystal packing come from H⋯O/O⋯H, H⋯H, H⋯N/N⋯H, and H⋯C/C⋯H interactions. In addition, FT-IR provided information on the functional groups involved in the coordination, while the decomposition patterns of both CPs were evaluated by TGA. Electrochemical studies conducted in a saline environment showed that CP1 exhibits superior hydrogen evolution reaction (HER) kinetics compared to CP2, as evidenced by a higher exchange current density and a lower Tafel slope. Density functional theory calculations and experimental bandgap measurements provided a deeper understanding of the electronic properties influencing the electrochemical behavior. The results highlight the potential of CP1 as an efficient catalyst for HER under saline conditions.

## 1. Introduction

Triazole compounds are five-membered aromatic heterocycles with three nitrogen atoms, making them strongly electron-deficient systems [1]. In a five-membered ring, a maximum of two types of positional arrangement of nitrogen atoms leads to the formation of two substantial isomers, namely, 1,2,3-triazole and 1,2,4-triazole [2]. The organic synthesis of triazoles and their fused heterocyclic derivatives have received significant attention in recent years mainly because they have a wide range of biological and pharmacological activities, such as antifungal, antimicrobial, antituberculosis, antibacterial, anti-inflammatory, anticonvulsant, antimalarial, and anticancer activities [3,4,5]. On the other hand, coordination polymers (CPs) regularly attract considerable research attention because they have proven to be promising materials for various applications, including heterogeneous catalysis, gas storage, and photoluminescence, among others [6,7]. In particular, the luminescence properties of these complexes are closely related to the structural and electronic characteristics of their ligands [8,9].

When triazoles are used as ligands in forming 2D-CPs, the products formed expand their properties and also possess a wide range of biological and pharmacological activities [10,11,12]. In particular, 1,2,3-triazole derivatives have been used to form CPs with different properties and applications [13,14,15]. For example, a new 2D CP based on a 1,2,3-triazole linker and La (III) ions has been developed, which is an efficient catalyst for the oxidation of olefins [16]. Additionally, novel 1,2,3-triazole-based ligands, Zn (II) and Cd(II) CPs, have been studied for their structural, thermal, computational, and luminescent properties [17].

As part of our continued efforts in the synthesis, structural characterization, and determination of the photophysical properties of triazole ligands and CP derivatives [17,18,19,20,21], we present here the synthesis, spectroscopic analysis, single-crystal structures, Hirshfeld surface analysis, electrochemical properties, and DFT calculations of CP1 and CP2 materials. The findings from DFT calculations, combined with experimental bandgap and electrochemical measurements, provide insight into the superior electrochemical performance of CP1 in hydrogen evolution.

## 2. Results and Discussion

### 2.1. Synthesis of NaL1

**Scheme 1:**
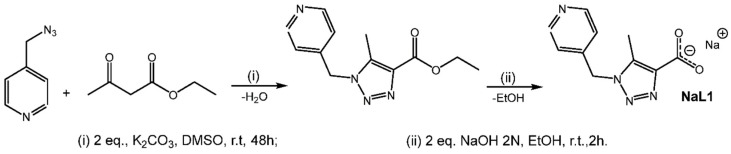
Synthetic route of NaL1 ligand.

The ester precursor of the organic ligand NaL1 was prepared according to standard methods reported in the literature [12,13] (Scheme 1).

### 2.2. Syntheses of CP1 and CP2

**Scheme 2:**
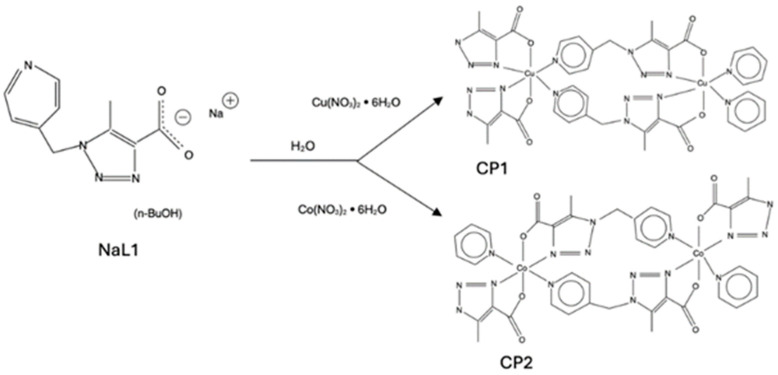
Synthetic routes of CP1 and CP2.

CPs were synthesized following a previously reported methodology (Scheme 2) [22]. Single crystals of CP1, [Cu(C_10_H_13_N_4_O_4_)_2_]_∞_ and CP2 [Co(C_10_H_9_N_4_O_4_)_2_]_∞_, suitable for X-ray diffraction were obtained by slow diffusion methods. The CP1 and CP2 compounds are blue and pale pink, respectively. Both compounds present stability in air and are insoluble in protic and aprotic solvents due to their polymeric composition.

### 2.3. Powder X-Ray Diffraction

The powder X-ray patterns of both compounds (Figure S1) were indexed in monoclinic cells and were corroborated by the X-ray single-crystal results. Figure S1 shows the good fit between the observed and calculated patterns. These results verify that the single crystals studied were representative of the bulk samples and that the powers and single crystals had the same structural phase.

### 2.4. Single-Crystal X-Ray Diffraction

Table 1 summarizes all the compounds’ crystal data, intensity data collection, and refinement details. X-ray crystal structure determinations and CIF files containing tables of crystallographic parameters, bond lengths, and bond angles, as well as a list of structural factors, have been deposited in the Cambridge Crystallographic Data Center (CCDC; nos. 2377095 and 2377094) and are freely available upon request from the following website: www.ccdc.cam.ac.uk/data_request/cif (accessed on 20 February2025).

**Figure 1:**
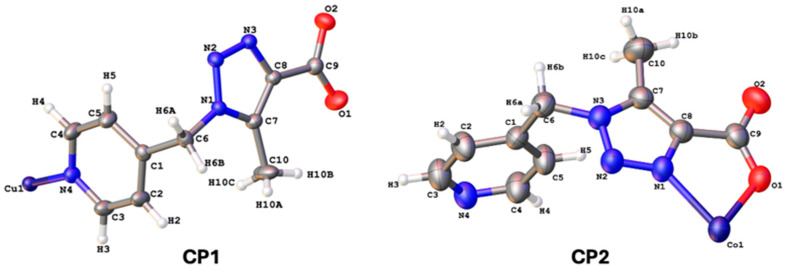
Asymmetric units for each compound with anisotropic ellipsoid representations, together with atom labeling schemes. The ellipsoids are drawn at a 30% probability level, and hydrogen atoms are depicted as spheres with arbitrary radii. The water molecules were omitted for clarity.

The title compounds crystallize in the monoclinic space groups *C2/c* (CP1) and *P2_1_/n* (CP2) with two molecules of the triazole linked to the metal atom and two water crystallization molecules for CP1. Figure 1 shows the molecular conformation and atom labeling of the title compounds. In the structures, all bond distances and angles are normal [23] and agree with the average values in similar entries found in the Cambridge Structural Database CSD, version 5.45, November 2023 [24].

**Figure 2:**
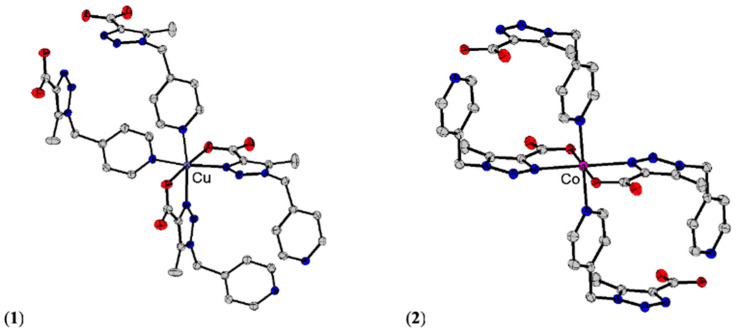
View of local coordination geometry at the Cu(II) and Co(II) centers for both compounds showing the cis and trans configurations. The hydrogen atoms were omitted for clarity. Red = Oxygen; Gray = Carbon; Blue = Nitrogen.

Figure 2 shows the Cu(II) and Co(II) ion coordination spheres of CP1 and CP2. The bond distances and angles are similar to those reported for related compounds, with N_4_O_2_ donors set as representative examples [25]; see Table 2. For CP1, the Cu(II) ion is set at the inversion center. Two nitrogen symmetry-related atoms of the triazole and pyridyl fragments build the equatorial plane around the Cu(II) ions. Meanwhile, the central Cu(II) ion is also coordinated by two oxygen symmetry-related atoms from carboxylate fragments at the axial positions. This gives rise to its distorted octahedral geometry, with the cis-N_4_O_2_ configuration consistent with the expected influence of the Jahn–Teller effect.

The compound CP1 is isostructural with the recently reported Zn(II) CP [22]. For CP2, the Co(II) ion lies on an inversion center. Two nitrogen symmetry-related atoms of the triazole and pyridyl fragments build the equatorial plane around the Co(II) ions. Meanwhile, the central Co(II) ion is also coordinated by two oxygen symmetry-related atoms from carboxylate fragments at the axial positions. This gives rise to its octahedral geometry with the trans-N_4_O_2_ configuration.

**Figure 3:**
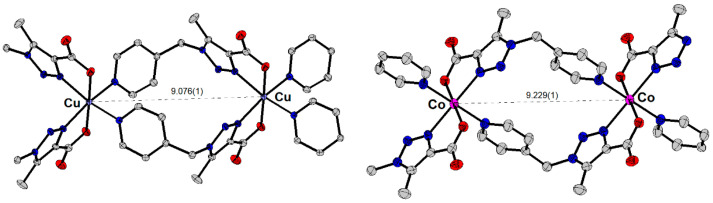
Polymeric metallocyclic complexes for CP1 and CP2, with the metal–metal distances shown. Red = Oxygen; Gray = Carbon; Blue = Nitrogen.

Both compounds display 1D zigzag chain structures. The structural analysis also revealed the presence of [2 + 2] polymeric metallocycle complexes with two ligand molecules coordinated to a pair of symmetry-related Cu(II) or Co(II) ions, resulting in the formation of metallocyclic rings formed in a rhomboid fashion in both compounds, with metal–metal separation distances in the rhomboid mean planes Cu∙∙∙Cu 9.076 (1) Å and Co∙∙∙Co 9.229 (1) Å, respectively (Figure 3).

For CP1, the crystal structure is stabilized by two intramolecular hydrogen bonds involving a C atom from the methyl group, O atoms of the carboxylate fragment, and water molecules, viz., C10−H10b∙∙∙O1 (H10b∙∙∙O1 2.53Å, C10−O1 3.17Å, and C10−H10b∙∙∙O1 123.7°) and C10−H10a∙∙∙O4 (H10a∙∙∙O4 2.68Å, C10−O4 3.56Å, and C10−H10a∙∙∙O4 153.3°). For CP2, the crystal structure is stabilized by one intramolecular hydrogen bond involving a C atom from the pyridine ring and an N atom of the triazole fragment, viz., C4−H4∙∙∙N2 (H4∙∙∙N2 2.77Å, C4−N2 3.42Å and C4−H4∙∙∙N2 127.5°) and C10−H10b∙∙∙O2 (2.60Å, C10−O2 3.26Å and C10−H10b∙∙∙O2 125.6°). For CP1, the hydrogen bond interaction between the triazole and pyridine fragments cannot be observed due to the cis N_4_O_2_ configuration. In CP1, the water molecules link a 1D polymeric chain, forming a 2D supramolecular aggregate. A 1D polymeric chain is linked to the neighboring chain by hydrogen bond interactions, O4−H4a∙∙∙O2 (C-O) and O4−H4b∙∙∙O1 (C=O), generating an $R_{4}^{4}(12)$ centrosymmetric ring labeled A and an $R_{4}^{4}$(30) ring labeled B [26]. These two types of rings alternate in an ABAB fashion to form a two-dimensional supramolecular aggregate. These kinds of interactions are reported in other compounds, generating extended 2D or 3D supramolecular networks through intermolecular hydrogen bonding [27,28,29].

**Figure 4:**
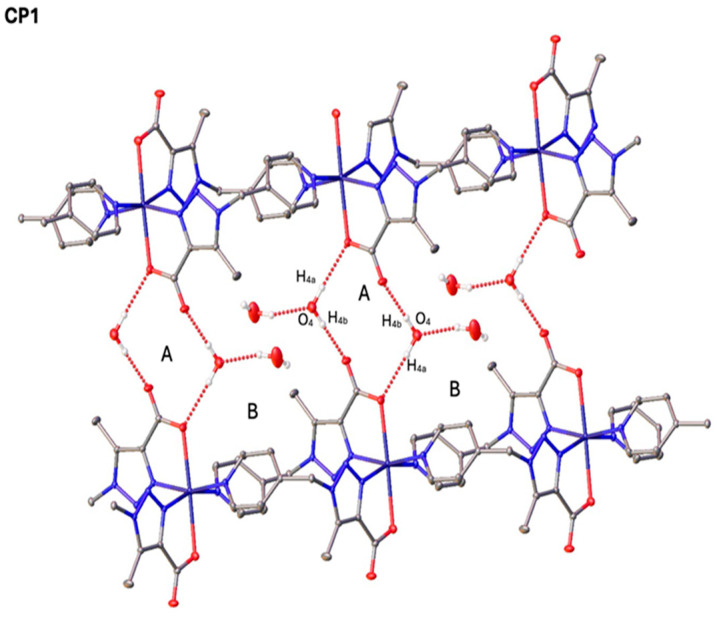

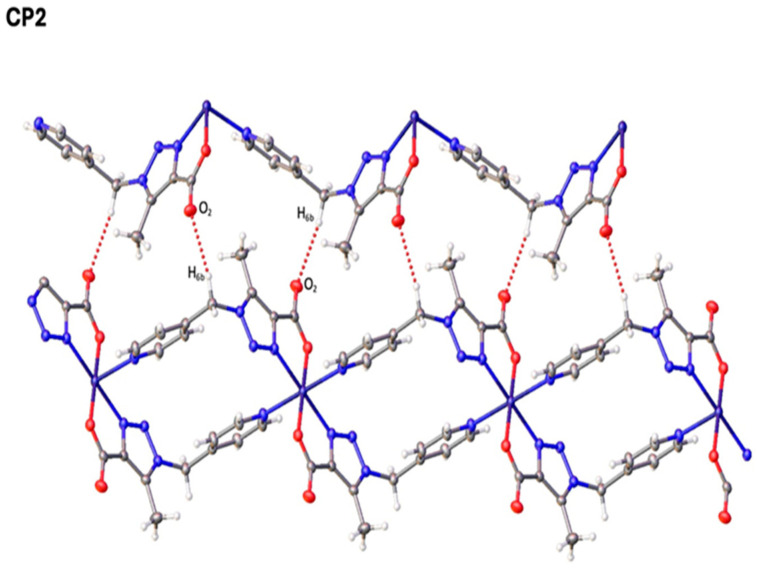
The 2D networks induced by hydrogen bond interactions for CP1 and CP2. For CP1, centrosymmetric $R_{4}^{4}(12)$ and $R_{4}^{4}$(30) rings (labeled A and B) alternate in an ABAB fashion, while for CP2 non-centrosymmetric $R_{2}^{2}(17)$ rings form a two-dimensional supramolecular aggregate.

In CP2, the 1D zigzag polymeric chain is linked to the neighboring chain by C6−H6b∙∙∙O2 hydrogen bond interactions generating an $R_{2}^{2}(17)$ non-centrosymmetric ring labeled A [26]; see Figure 4 and the Supplementary Materials (Tables S1 and S2).

### 2.5. Fourier-Transform Infrared Spectroscopy (FTIR)

The FT-IR spectra obtained from the ligand and CP1 and CP2 allowed a qualitative characterization of the structures; the most important bands are summarized in Table S3 and Figure S2 (see the Supplementary Materials). The infrared spectrum of the ligand shows a band at 3475 cm^−1^ associated with the asymmetric and symmetric ν(OH) stretching vibrational modes of water molecules. The ν(CH) stretching vibration of pyridine was observed at 3047 cm^−1^. The intermediate peak center at 1563 cm^−1^ of high intensity may be due to the overlap of the ν(C=N) and ν(C=C) stretching modes of pyridine, in addition to the ν(N=N) stretching vibration of triazole. The characteristic bands of the carboxylate groups were found at 1596 and 1212 cm^−1^. The ν(C=N) stretching vibration of the triazole is characterized by a strong sharp band located at 1443 cm^−1^. The C-C and C-N pyridine and N-N triazole stretching vibrations range from 1365 to 1005 cm^−1^ [30]. For CP1 and CP2, the same bands of the free ligand can be observed, slightly shifted in some cases towards higher frequencies. The vibrational bands of the ν(C=O) and ν(C-O) groups of the coordinated carboxylate can be observed at 1613 and 1218 cm^−1^ for CP1 and 1611 and 1218 cm^−1^ for CP2. The ν(C=N) and ν(N=N) stretching vibrations of the triazole ring in both compounds are in the range of 1566–1503 cm^−1^, while the C-C, C-N, and N-N stretching vibrations are in the range of 1600–1000 cm^−1^.

### 2.6. Thermal Stability Studies (TGA)

The thermal studies of CP1 and CP2 were analyzed through TGA in the temperature range of 25 to 1000 °C at a heating rate of 10 °C/min in a nitrogen environment. CP1 was stable up to ca. 50 °C; after this temperature, the progressive decomposition of the complex occurred. The organic ligand completely decomposed at 350 °C, losing 55% of the weight. The 45% black residue remained at the end of the heating process, which contained CuO and carbonaceous matter. Similarly, CP2 started decomposing from 60 °C, and complete decomposition of the ligand took place at 450 °C, with a weight loss of 70%. The remaining black residue contained CoO and carbonaceous materials (see the Supplementary Materials, Figure S5).

### 2.7. Hirshfeld Surface Analysis

The nature of the intermolecular interactions in the title compounds was computed using the CrystalExplorer 17.5 software [31] using Hirshfeld surface analysis [32] and two-dimensional fingerprint plots [33]. The dnorm plots were estimated via calculations of the external (de) and internal (di) distances to the nearest nucleus (Figure 4). The red areas on the surfaces reflect short contacts and negative dnorm values, which correspond to the hydrogen···hydrogen, O···H, N···H, and C···H contacts explained above (see the Supplementary Materials, Table S4). The plots of the Hirshfeld surfaces confirm the presence of non-covalent interactions.

**Figure 5:**
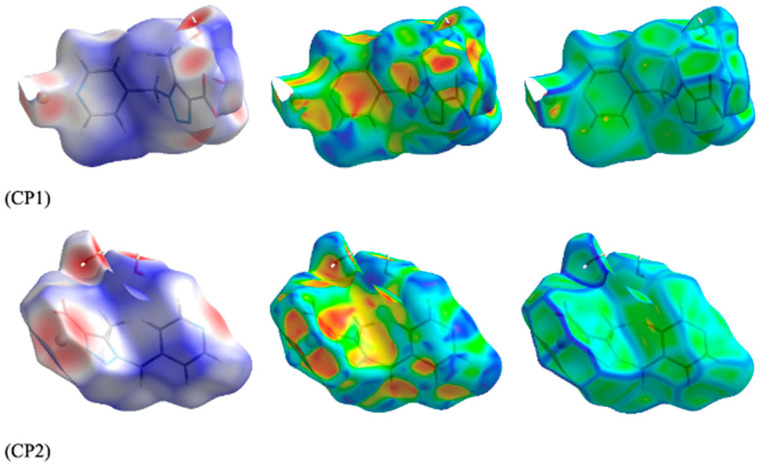
Views of the three-dimensional Hirshfeld surfaces of **CP1** and **CP2**, with dnorm plots, shape indexes, and curvedness.

The shape index of a Hirshfeld surface is a tool for visualizing π···π stacking interactions by the presence of adjacent red and blue triangles. Figure 5 indicates that there are no adjacent red and/or blue triangles and no π···π interactions in the crystals of the title compounds, as was indicated by the X-ray crystal structure analysis.

Fingerprint plots were produced for CP1 and CP2 (see the Supplementary Materials, Figure S6). The overall two-dimensional fingerprint plot for CP1 and those delineated into O··H/H···O (37.4%), H···H (24.9%), N···H/H···N (9.71%), C···H/H···C (9.4%), and Cu···N/N···Cu (6.8%) contacts are shown in Figure 5. For CP2, similar hydrogen bonds were observed.

### 2.8. Density Functional Theory (DFT) Calculations

**Figure 6:**
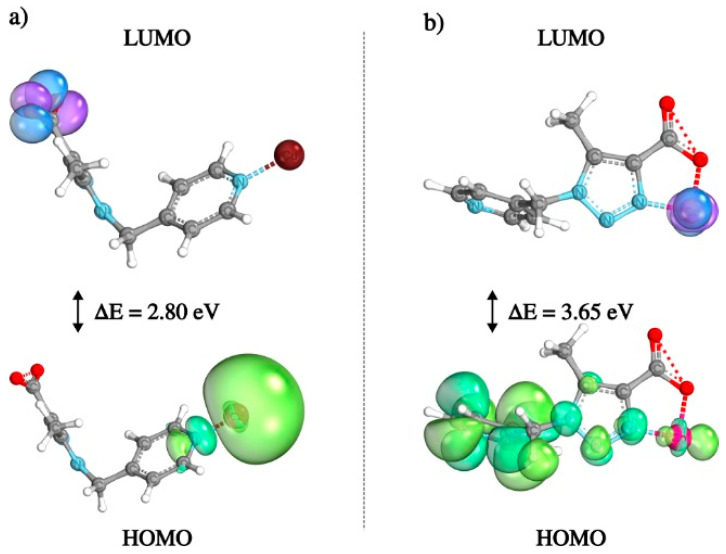
Visualizations of the highest occupied (HOMO) and lowest unoccupied (LUMO) molecular orbitals for CP1 (**a**) and CP2 (**b**).

Figure 6 illustrates the frontier molecular orbitals (FMOs) of CP1 and CP2, calculated using DFT. The distributions of the highest occupied molecular orbitals (HOMOs) and the lowest unoccupied molecular orbitals (LUMOs) reveal distinctive electronic characteristics for each compound. In CP1, the HOMO is primarily localized around the copper center and adjacent ligands, while the LUMO is centered around the C=O group, resulting in an energy gap of 2.80 eV. Conversely, CP2 shows a more delocalized HOMO across the ligand framework, with the LUMO more localized around the cobalt center. This configuration leads to a considerably larger energy gap (ΔE = 3.65 eV) in CP2 compared to CP1, indicating enhanced electronic stability. Also, this result suggests a higher reactivity for CP1 than for CP2. These differences align with the observed crystallographic and supramolecular interactions, highlighting the influence of metal–ligand coordination on the electronic properties of these materials.

The experimental bandgaps of CP1 and CP2 were investigated by a UV/vis diffuse-reflectance measurement method at room temperature. The results showed Eg (bandgap energy) values of 2.63 and 3.62 eV for CP1 and CP2, respectively (Figure S7). The lower HOMO-LUMO energy gap of CP1 compared to CP2, obtained from both theoretical and experimental analyses, suggests enhanced charge transfer efficiency. This electronic characteristic is expected to play a role in the electrochemical behavior of CP1, as has been described for other molecules [34,35,36] and as will be discussed in the following section.

### 2.9. Electrochemical Analysis of Materials

**Figure 7:**
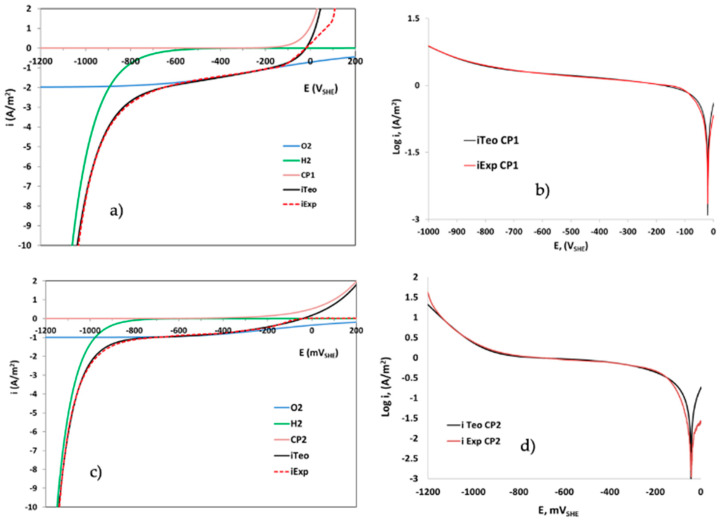
(**a**–**c**) Linear polarization curves and (**b**–**d**) Tafel polarization curves for CP1 and CP2 electrodes in aerated 0.5 M NaCl, 2 mV/s and 0 rpm, considering a kinetic order equal to 0.5.

The kinetic investigation was conducted through non-linear fitting of experimental polarization data, employing the superposition model and mixed potential theory, as detailed in our prior studies [37,38,39], focusing on charge transfer, mass diffusion, and passivation mechanism controls. Figure 7 offers crucial insights into the electrochemical–kinetic behavior of the 2 and 1 powders during the sub-cathodic process in 0.5 M NaCl solution. Figure 7a–c depict the results of linear sweep voltammetry (LSV) experiments, with a potential window ranging from −1200 to 0 mV_SHE_.

Figure 7a–c present the superposition model output curves for CP1 and CP2 electrodes in aerated 0.5 M NaCl at 2 mV/ and 0 rpm derived from linear sweep voltammetry (LSV) experimental data. The potential window applied during the experiments ranged from −1200 to 0 mV/SHE. The Tafel slopes for the cathodic potential range, in which the hydrogen evolution reaction (HER) mechanism occurs, were −230 and −181 mV/dec, while during ORR the current limits were −1.996 and −0.998 A/m^2^ for the CP1 and CP2 electrodes, respectively. The resistance of the CP1 and CP2 electrodes in contact with the NaCl solution revealed corrosion potential $\left(E_{corr}\right)$ values of −19.995 and −43.482 mV/SHE, respectively. Additionally, the current corrosion ($i_{corr}$) values were 0.744 and 0.388 A/m^2^ for the CP1 and CP2 electrodes, respectively. These $E_{corr}$ and $i_{corr}$ values suggest a high corrosion resistance, supported by the performance of the Evans curve depicted in Figure 6a,b and the electrochemical kinetic parameters for HER, ORR, and CP_m_OR presented in Table S5 (see the Supplementary Materials), which displays the essential corrosion and kinetic parameters derived from employing the superposition model, leveraging Equations (1)–(4). These parameters were delineated concerning their associated controls, encompassing charge transfer, mass diffusion, and dissolution mechanisms. This thorough analysis offers valuable insights into the dominant factors and mechanisms shaping the corrosion behavior within the examined system [38,39,40,41]. The electrochemical results are consistent with the electronic properties derived from both the DFT calculations and experimental UV/vis spectroscopy. The lower HOMO-LUMO gap of CP1 (2.80 eV theoretical, 2.63 eV experimental) facilitates charge transfer processes, contributing to its higher exchange current density and lower Tafel slope. This finding suggests that CP1 exhibits superior catalytic performance for HER compared to CP2, where the larger bandgap (3.65 eV theoretical, 3.62 eV experimental) indicates greater electronic stability but reduced reactivity.

The results depicted in Table S5 (see the Supplementary Materials), utilizing the superposition model based on mixed potential described in equations 1 to 4, reveal noticeable variations in parameters such as $i_{0,H_{2}}$ and $i_{0,{CP}_{m}}$ for the CP1 and CP2 electrodes. Particularly noteworthy is the $i_{0,H_{2}}$ association with HER, which exhibited intriguing variability when the CP1 and CP2 electrodes interacted with the saline solution at the cathodic potentials. At the same time, the $i_{0,{CP}_{m}}$ experiment exhibited similar variations in anodic potentials. $i_{0,O_{2}}$ indicated rapid reaction processes occurring at the electrode–electrolyte interface, a critical factor for optimal performance of this electrochemical device.

## 3. Materials and Methods

### 3.1. Synthesis of NaL1

The compound precursor, ethyl 5-methyl-1-(pyridin-3-ylmethyl)-1H-1,2,3-triazole-4-carboxylate, of the organic ligand, NaL1, was prepared according to standard methods reported in the literature [17,18]. The ester was saponified with NaOH (2 eq.) solution in a minimum amount of methanol to generate the corresponding carboxylate sodium salt compound, NaL1, yielding 80%.

### 3.2. Syntheses of CP1 and CP2

A 0.115 mmol solution of Cu(NO_3_)_2_∙6H_2_O or Co(NO_3_)_2_∙6H_2_O in n-butanol (5 mL) was slowly added over an aqueous solution of NaL1 (0.230 mmol, 5 mL) following the procedure reported in [21]. The slow diffusion of the solution of reactants in immiscible solvents (n-BuOH/H_2_O, stored at room temperature) for two weeks resulted in the formation at the interphase of single crystals of CP1 and CP2 suitable for XRD analysis. The crystals were collected by hand and air-dried. Yields: 70 and 60%, respectively (Scheme 1).

### 3.3. Powder X-Ray Diffraction

The powder X-ray diffraction data for CP1 and CP2 were collected at room temperature in a Bruker D8 diffractometer with CuKα radiation (λ = 1.5418 Å). The diffractometer was operated at 40 kV and 30 mA in θ/θ reflection mode. The powder patterns were scanned from 5° to 55° of 2θ, with a step size of 0.02° and a counting time of 1 s per step. For each compound, a Rietveld refinement [31] was carried out using the Fullprof program [32] to check the structural parameters.

### 3.4. Single-Crystal X-Ray Diffraction

Suitable single crystals of CP1 and CP2 were selected and measured. Diffraction data were collected at 293 (2) K on a Bruker D8 Venture diffractometer equipped with a Photon-III C14 detector using graphite monochromated MoKα (λ = 0.71073 Å) radiation (Bruker Co.; Billerica, MA, USA). The diffraction frames were integrated using the Apex4 package [33] and were corrected for absorptions with Sadabs [23]. The crystal structures of the title compounds were solved by intrinsic phasing [33] using the Olex2 software V1.5, [25] and refined with full-matrix least-squares methods based on F2 (Shelxl) [42]. All the non-hydrogen atoms were refined with anisotropic displacement parameters. The hydrogen atoms were included from calculated positions and refined riding on their respective carbon atoms with isotropic displacement parameters. All geometrical calculations were performed using the program Platon [43]. Disordered water molecules located inside voids of CP2 were not modeled, and the disordered density was taken into account using the solvent mask calculations implemented in the Olex2 software.

### 3.5. Fourier-Transform Infrared Spectroscopy (FTIR)

FT-IR spectra were recorded on a Thermo Electron Corporation model Nicolet Avatar 330 (Thermo Scientific, Waltman, MA, USA) with KBr disks in the 4000 to 400 cm^−1^ range.

### 3.6. Thermal Stability Studies (TGA)

TGA measurements were carried out on an STA 448 Jupiter F3-type simultaneous thermal analyzer (Netzsch, Selb, Germany). For TGA, 5.0 mg quantities of the samples were used as microcrystalline powders. The used sample cells were pans. The parent reagents were heated up to 400 °C at a heating rate of 10 °C min^−1^ under nitrogen flow at 20 mL min^−1^.

### 3.7. Hirshfeld Surface Calculations

Hirshfeld surfaces and fingerprint plots were calculated for the title compounds based on the crystallographic information file (CIF) using the CrystalExplorer 17.5 software [44,45]. The 2D fingerprint plots were displayed using the standard 0.6–2.6 Å view, with the *de* and *di* distance scales displayed on the graph axes [46]. The function *dnorm* is a ratio enclosing the distances of any surface point to the nearest interior (di) and exterior (de) atom and the van der Waals (vdW) radii of the atoms. Hirshfeld surfaces were obtained using a high surface resolution, with the three-dimensional dnorm surfaces mapped over a fixed color scale of −0.728 (red) to 1.428 (blue). The points which make small contributions to a Hirshfeld surface are colored blue, and the points which make the greatest contributions are colored red. Shape index surfaces are useful for the analysis of π interactions because they provide qualitative measures of surfaces. Hirshfeld surfaces in curvedness studies show how flat a surface is; relatively flat regions are shown in green and are separated by dark-blue edges of positive curvature. These flat regions allow the identification of regions susceptible to interactions of the π···π types face–face or edge–edge that take place between conjugated systems of aromatic rings and double bonds. Fingerprint plots enable observation of contributions from different interaction types, and provide a valuable quantitative analysis of the intermolecular interactions occurring in a crystal structure. For the generation of fingerprint plots, the bond lengths of hydrogen atoms involved in interactions were normalized to standard neutron values (C-H = 1.083 Å, N-H = 1.009 Å, and O-H = 0.983 Å) [47].

### 3.8. Density Functional Theory (DFT) Calculations

DFT calculations were performed using the ORCA software package [48,49]. Structural optimizations of the coordination polymers were performed using the PBE0 functional combined with the def2-TZVP basis set. Harmonic vibrational frequency calculations were subsequently conducted to confirm the absence of imaginary frequencies in the optimized geometries. Single-point energy calculations were then performed using PBE0-D3BJ/def2-TZVP for the Co coordination polymer and wB97X-D3/def2-TZVP for the Cu coordination polymer in order to carry out frontier molecular orbital (FMO) analysis and simulate the infrared spectra of the complexes. These methods were selected for their computational efficiency and suitability in accurately capturing the electronic properties of the coordination polymers.

### 3.9. Electrochemical Measurements of CP1 and CP2 Electrodes

The procedure for electrochemical measurements was devised to investigate the kinetics of partial electrochemical reactions of CP1 and CP2 electrodes immersed in 0.5 M NaCl solutions, with a particular focus on HERs (hydrogen evolution reactions) and ORRs (oxidation reactions). Each carbon-paste electrode was prepared by blending 0.2 g of CP1 and CP2 powder with 0.25 g of graphite powder and 0.2 cm^3^ of paraffin wax to achieve a uniform paste. These pastes were densely packed into a plastic teflon sheath (4 mm in diameter and 10 mm in length) with adequate perforations to maintain electrical contact via a copper wire and the CP powders in a rotating disc electrode (RDE) system. Current density-versus-potential curves were obtained through linear sweep voltammetry (LSV) measurements, utilizing a BASI/RDE-2 rotating electrode interface connected to an Epsilon potentiostat/galvanostat in a conventional 3-electrode cell setup. Here, the carbon-paste electrode with CP1 and CP2 powders served as the working electrode, platinum wires served as the counter electrode, and Ag/AgCl (4M KCl Sat.) served as the reference electrode (RE). All potentials reported were referenced to the standard hydrogen electrode (SHE). The experimental protocol for polarization data followed a prior study within a potential range of −1200 to 0 mV/SHE. Before each run, the CP1 and CP2 electrodes were held at −1400 mV for 60 s.

### 3.10. Electrochemical and Kinetic Analysis

The kinetic analysis was conducted by employing non-linear fitting to experimental polarization curves derived from LVS (linear scan voltammetry) measurements. We adopted the superposition model rooted in mixed potential theory, as outlined in our prior research on mass diffusion, charge transfer, and passivation mechanism controls. The non-linear fitting methodology incorporates a set of kinetic expressions (Equations (1)–(4)), which relate the total current density (i) to the partial current densities for the ORR ($i_{O_{2}}$), HER ($i_{H_{2}}$), and CP_m_OR, where m is Cu ($i_{Cu}$) or Co ($i_{Co}$).

(1) $$i=i_{O_{2}}+i_{H_{2}}+i_{{CP}_{m}}$$

(2) $$i_{O_{2}}=i_{0,O_{2}}{\left(1-\frac{i_{O_{2}}}{i_{l,O_{2}}}\right)}^{p}e^{\left(\frac{-2.303\eta_{O_{2}}}{t_{O2}}\right)}$$

(3) $$i_{H_{2}}=i_{0,H_{2}}e^{\left(\frac{-2.303\eta_{H_{2}}}{t_{H2}}\right)}$$

(4) $$i_{{CP}_{m}}=i_{0,{CP}_{m}}e^{\left(\frac{2.303\eta_{{CP}_{m}}}{t_{{CP}_{m}}}\right)};\;m:\;Cu\;or\;Co$$

where $i_{0{,O}_{2}}$, $i_{0,H_{2}}$, and $i_{0,{CC}_{m}}$ are the exchange current densities for the ORR, HER, and CP_m_OR (m: Cu or Co); $i_{l,O_{2}}$ is the limiting current density for the ORR; $\eta_{O_{2}}=E-E_{eqO_{2}}$, $\eta_{H_{2}}=E-E_{eqH_{2}}$, and $\eta_{{CP}_{m}}=E-E_{eq{CP}_{m}}$ are the overpotentials for the ORR, HER, and CP_m_OR; E is the electrochemical potential; $E_{eqO_{2}}$, $E_{eqH_{2}}$, and $E_{eq{CP}_{m}}$ are the equilibrium potentials for the ORR, HER, and CP_m_OR, respectively; $t_{O2}$, $t_{H2}$, and $t_{{CP}_{m}}$ are the Tafel slopes for the ORR, HER, and CP_m_OR, respectively; and p is the kinetic order for the ORR and is equal to 0.5. The kinetic parameters were calculated from the fitting to the experimental data [38,39,40,41].

## 4. Conclusions

Two new coordination polymers, CP1 and CP2, with the methyl-1-(pyridin-4-yl-methyl)-1H-1,2,3-tria-zole-4-carboxylate ligand have been prepared at the water/n-butanol interphase. Both compounds, CP1 and CP2, display 1D zigzag chain structures which further result in two-dimensional supramolecular networks due to O-H⋯O, C-H⋯O, and C—H···N hydrogen bond interactions. The structural analysis also revealed the presence of [2 + 2] polymeric metallocycle complexes. The fingerprint plots for CP1 and CP2 show that these interactions are similar (in percentages) (O···H/H···O percentages of 37.4 for CP1 and 35.2 for CP2). However, the values for the N···H/H···N and C···H/H···C interactions are different (9.7/14.9 and 9.4/14.1 for CP1 and CP2, respectively); these kinds of interactions make the supramolecular structures different. The *i*_0,*H*2_ is higher for CP2 than for CP1, indicating that CP1 exhibits more favorable electrochemical kinetics for HER in contact with 0.5M NaCl. On the other hand, the Tafel slope of CP1 is less negative compared to the CP2 material, likely due to a lower energy barrier. More negative Tafel slope values indicate that the material requires more energy to increase the reaction rate. Regarding the ORR kinetics, it can be interpreted that CP1 exhibits better performance than CP2 in the range of −600 to −100 mV/SHE. Consequently, it can be interpreted that CP1 may increase the rate of O_2_ reduction. The findings from the DFT calculations, combined with the experimental bandgap measurements, provide insight into the superior electrochemical performance of CP1. The smaller HOMO-LUMO gap facilitates charge transfer, leading to enhanced HER kinetics and lower Tafel slopes, making CP1 a more efficient catalyst in saline environments compared to CP2.

## Figures and Tables

**Table 1:** Crystal data, data collection, and structural refinement of the title compounds (CP1 and CP2).

	CP1	CP2
CCDC number	2377095	2377094
Empirical formula	C_20_H_26_CuN_8_O_8_	C_20_H_18_CoN_8_O_4_
Formula weight	570.04	493.35
Temperature/K	293	293
Crystal system	Monoclinic	Monoclinic
Space group	*C2/c* (No 15)	*P2_1_/n* (No 14)
a/Å	19.5486 (16)	10.9360 (14)
b/Å	9.0762 (6)	9.2289 (10)
c/Å	14.4365 (11)	13.3890 (17)
β/°	102.627 (3)	106.044 (4))
Volume/Å^3^	2499.5 (3)	1298.7 (3)
Z′	1	1
Z	4	2
ρ_calc_g/cm^3^	1.515	1.262
μ/mm^−1^	0.935	0.699
F(000)	1180	507
Crystal size/mm^3^ Color	0.11 × 0.21 × 0.30 Blue prism	0.15 × 0.20 × 0.38 Light pink
Radiation	MoKα (λ = 0.71073)	MoKα (λ = 0.71073)
2Θ range for data collection/°	2.50 to 30.5	2.72 to 27.1
Index ranges	−27 ≤ h ≤ 27, −11 ≤ k ≤ 12, −20 ≤ l ≤ 20	−14 ≤ h ≤ 13, −10 ≤ k ≤ 11, −17 ≤ l ≤ 17
Reflections collected	23,651	21,181
Independent reflections	3811 [R_int_ = 0.058]	2864 [R_int_ = 0.066]
Data/restraints/parameters	3811/0/175	2864/0/153
Goodness-of-fit on F^2^	1.034	1.26
Final R indexes [all data]	R_1_ = 0.0366, wR_2_ = 0.101	R_1_ = 0.066, wR_2_ = 0.2863
Largest diff. peak/hole/e Å^−3^	0.50/−0.50	0.80/−0.70

**Table 2:** Selected geometrical parameters (Å) for CP1 and CP2.

CP1		CP2	
Bond Distance (Å)			
Cu–O2 ^2^	2.404 (14)	Co1–O1	2.073 (4)
Cu–O2 ^1^	2.404 (14)	Co1–O1 ^1^	2.074 (4)
Cu–N3 ^1^	2.028 (13)	Co1–N1	2.124 (4)
Cu–N3 ^2^	2.028 (13)	Co–N1 ^1^	2.124 (4)
Cu–N4	2.032 (14)	Co1–N4 ^2^	2.174 (4)
Cu–N4 ^4^	2.032 (14)	Co–N4 ^3^	2.174 (4)

Symmetry codes: ^1^ 1 − x, 2 − y, 1 − z; ^2^ x, 1 + y, +z; ^3^ 1 − x, 1 − y, 1 − z; ^4^ 1 − x, +y, ½ − z.

## Data Availability

Data are contained within the article and the Supplementary Materials.

## Supplementary Materials

The following supporting information can be downloaded at: https://www.mdpi.com/article/10.3390/ijms26041671/s1.
