# Supplementary material for: Synthesis, Crystal Structures, Hirshfeld Surface Analysis, Computational Investigations, Thermal Properties, and Electrochemical Analysis of Two New Cu(II) and Co(II) Coordination Polymers with the Ligand 5-Methyl-1-(pyridine-4-yl-methyl)-1H-1,2,3-triazole-4-carboxylate
Source: Int J Mol Sci. 2025 Feb 15;26(4):1671. doi: 10.3390/ijms26041671 (PMC11855095; doi:10.3390/ijms26041671)
Supplement: Supplementary file 1 [file ijms-26-01671-s001.zip › ijms-3435819-supplementary.pdf]

Electronic Supplementary Information (ESI)

Synthesis, crystal structure, Hirshfeld surface analysis, computational investigations, thermal properties and electrochemical analysis, of two new Cu(II) and Co(II) coordination polymers with the ligand 5-methyl-1-(pyridine-4-yl-methyl)-1H-1,2,3-triazole-4-carboxylate

Markus Bergedahl [1], Pilar Narea [1], Jaime Llanos [2], Ruth Pulido [1,3], Nelson Naveas [3,4,5], Pilar Amo-Ochoa [6,7], Félix Zamora [6,,8], Gerzón E. Delgado [9], Felipe M. Galleguillos Madrid[10], Yasna León[1],Iván Brito [1*]

Contents

1. Supplementary Figure..2
2. Supplementary Table...7

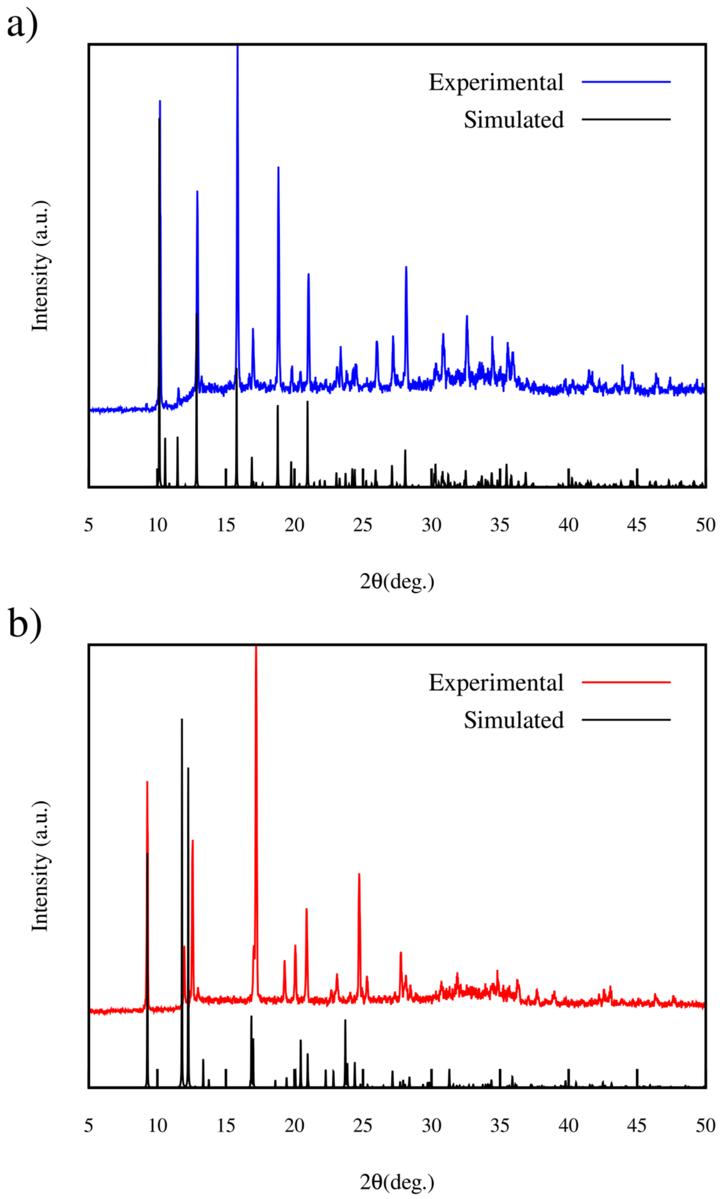

Figure S1 P-XRD of compounds CP1 simulated (black) and experimental (blue)and CP2 simulated (black) and experimental(red)

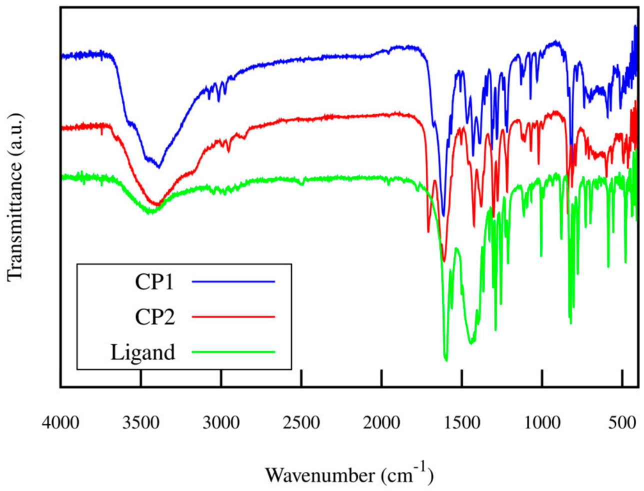

Figure S2. FT-IR spectra of Ligand, CP1 and CP2

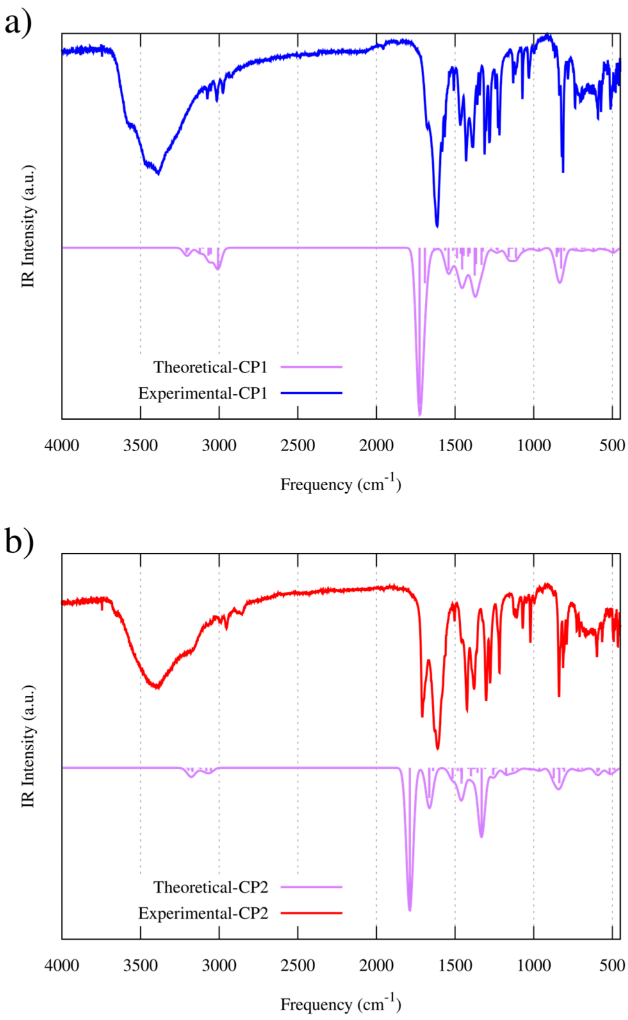

Figure S3. Experimental and theoretical IR spectra of (a) CP1 and (b) CP2.

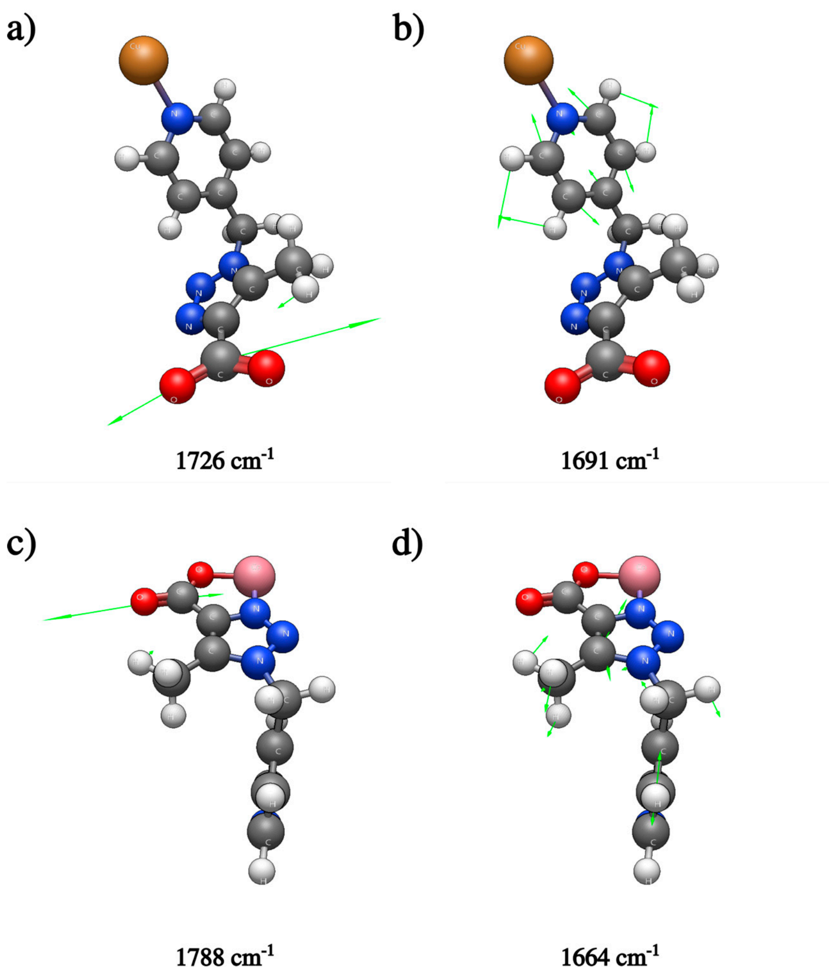

Figure S4. Main atomic displacements for representatives IR vibrations modes of (a-b) CP1 and (c-d) CP2 calculated by DFT.

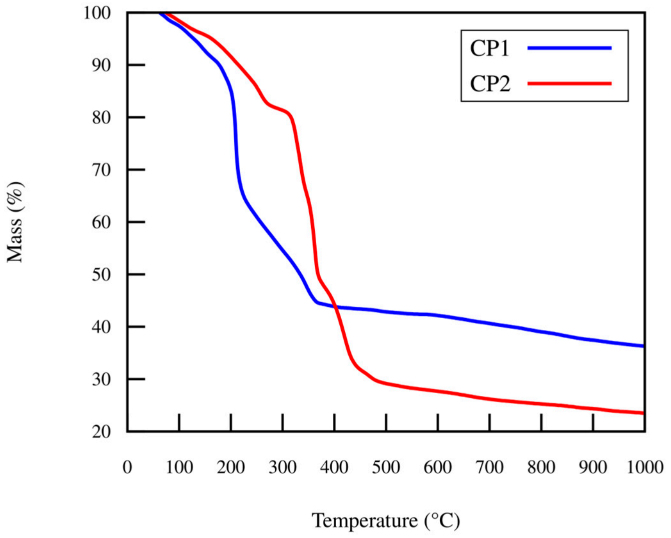

Figure S5. Thermograms of the CP1 and CP2 were obtained from 50 to 100°C under a nitrogen atmosphere at a heating rate of 10° C/min.

(1)

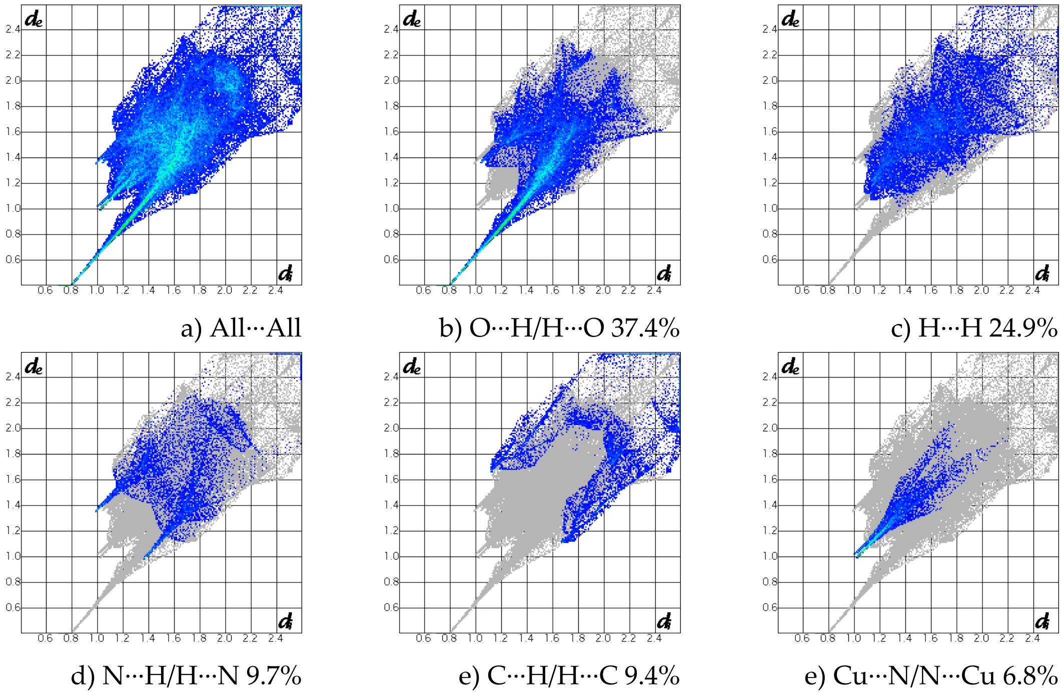

(2)

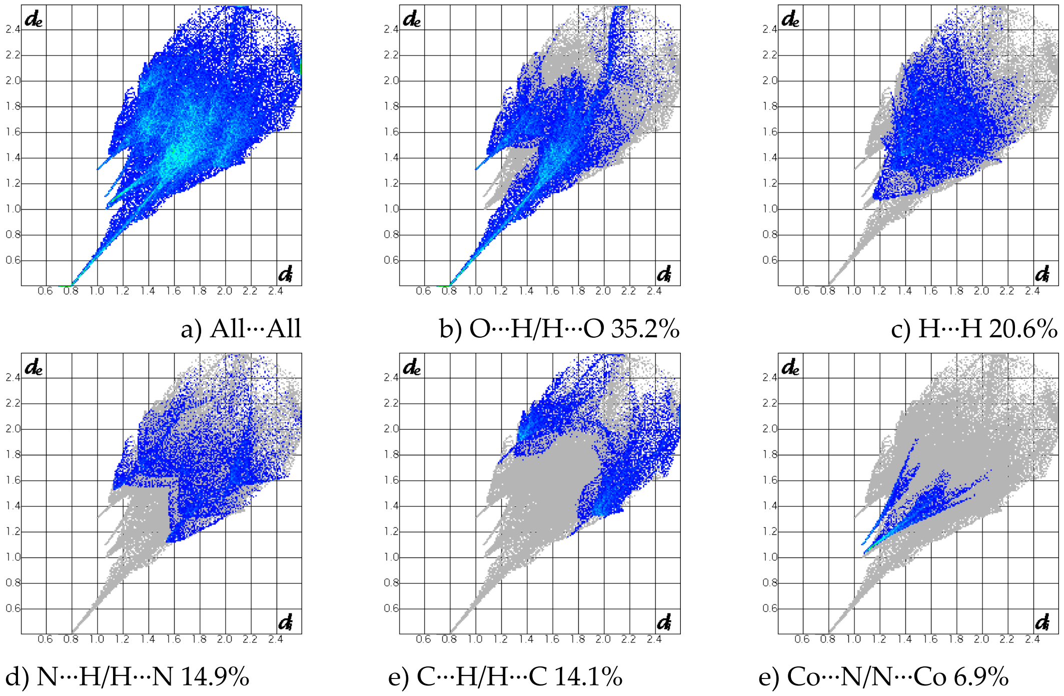

Figure S6. The full two-dimensional fingerprint plots for CP1 and CP2.The *di* and *de* values are the closest internal and external distances (in Å) from given points on the Hirshfeld surface.

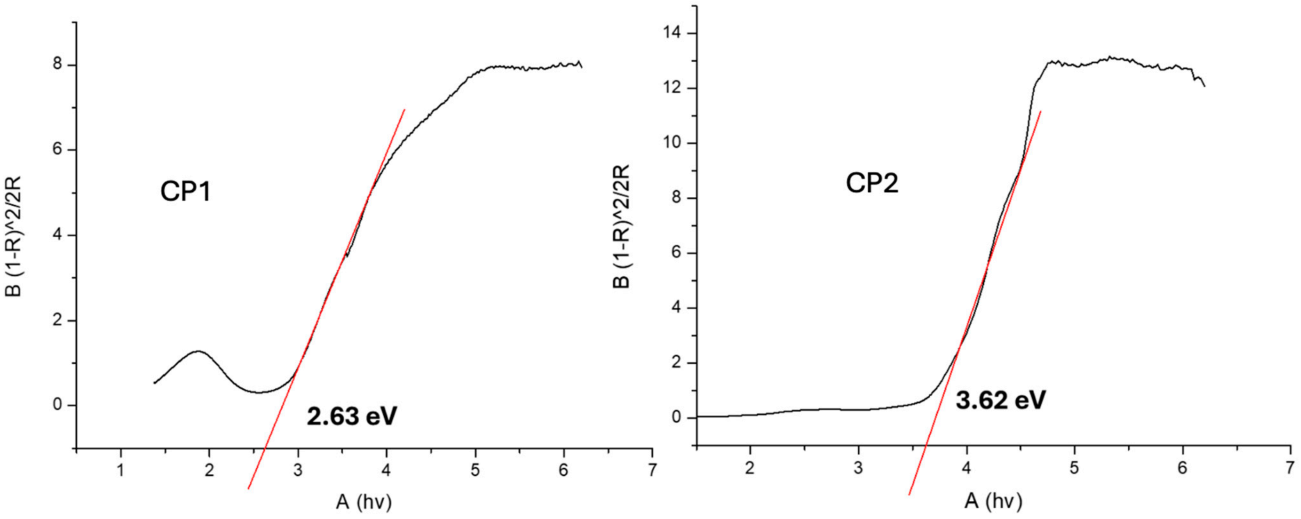

Figure S7. Band gap experimental value by diffuse reflectance for CP1 and CP2.

Table S1. Intra and intermolecular hydrogen bond for CP1 (Å, °)

D—H···A	D—H	H···A	D···A	D—H···A
C_{10}—H_{10b}···O_1	0.96	2.53	3.17 (3)	124
C_{10}—H_{10a}···O_4	0.96	2.68	3.56 (3)	153

Table S2. Intra and intermolecular hydrogen bond for CP2 (Å, °)

D—H···A	D—H	H···A	D···A	D—H···A
C_4—H_4···N_2	0.97	2.77	3.42 (7)	128
C_{10}—H_{10b}···O_2	0.97	2.60	3.26 (9)	126
C_6—H_{6b}···O_2^{i}	0.97	2.43	3.39 (7)	169

Symmetry code: (i) 3/2-x, -1/2+y, ½-z

Table S3. FT-IR bands of the Ligand and CP1 and CP2

**Compound**	**IR bands**					
**Ligand**	3464	3047	1596	1563	1443	1212
	νO-H	νC-H	νC=O	νC=N<br>ν N=N<br>νC=C	νC=N	νC-O
**CP1**	3392	3016	1613	1566	1466	1218
	νO-H	νC-H	νC=O	νC=N<br>νN=N<br>νC=C	νC=N	νC-O
**CP2**	3407	2954	1611	1503	1424	1218
	νO-H	νC-H	νC=O	νC=N<br>νN=N<br>νC=C	νC=N	νC-O

Table S4. Visualizations of the highest occupied (HOMO) and lowest unoccupied (LUMO) molecular orbitals for CP1 and CP2

Parameters	CP1	CP2
E_{LUMO} (eV)	-3.2774	-1.4791
E_{HOMO} (eV)	-6.0732	-5.1293
ΔE (eV)	2.7958	3.6502
$E_{LUMO} + E_{HOMO}$ (eV)	-9.3506	-6.6084
Electronegativity (χ) (eV)	4.6753	3.3042
Chemical Potential (μ) (eV)	-4.6753	-3.3042
Chemical Hardness (η) (eV)	1.3979	1.8251
Softness (S) (eV^{-1})	0.3577	0.274

Table S5. Kinetic and corrosion parameters calculated from experimental polarization curves for CP_mOR (where m is Cu or Co), immersed in NaCl solutions

Parameters	CP1	CP2
i_{0,H_2}, A/m^2	-0.996	-4.612
t_{H_2}, mV/dec.	-230	-181
i_{0,O_2}, A/m^2	0.0841	0.0206
i_{l,O_2}, A/m^2	-1.996	-0.998
i_{0,CC_m}, A/m^2	$1.93 \cdot 10^{-4}$	$2.75 \cdot 10^{-2}$
t_{CC_m}, mV/dec.	117	345
E_{corr}, mV/SHE	-19.995	-43.482
i_{corr}, A/m^2	0.744	0.388
